# Draft genome sequence data of *Vibrio cholerae* responsible for cholera outbreaks in Ethiopia

**DOI:** 10.1128/mra.00272-25

**Published:** 2025-06-23

**Authors:** Abebaw Bitew Kifilie, Molalegne Bitew, Aschalew Gelaw, Yitayih Wondimeneh, Abaysew Ayele, Dawit Hailu Alemayeh, Biruk Yeshitela, Ashenafi Alemu, Getachew Tesfaye Beyene, Baye Gelaw, Adane Mihret

**Affiliations:** 1 Department of Medical Microbiology, School of Biomedical and Laboratory Sciences, College of Medicine and Health Sciences, University of Gondar 362057, Gondar, Ethiopia; 2 Department of Medical Microbiology, College of Medicine and Health Sciences, Debre Markos University 241184, Debre Markos, Ethiopia; 3 Health biotechnology Directorate, Bio and Emerging Technology Institute of Ethiopia, Addis Ababa, Ethiopia; 4 Bacterial and Viral Disease Research Directorate, Armauer Hansen Research Institute 70605, Addis Ababa, Ethiopia; Wellesley College, Wellesley, Massachusetts, USA

**Keywords:** draft, genome, sequence, cholera, outbreak, Ethiopia

## Abstract

Cholera is an acute diarrheal disease caused by *Vibrio cholerae*. Low-income countries, including Ethiopia, have been attacked by repeated outbreaks. Here, we present the draft genome sequence of *V. cholerae* O1 strains isolated from clinical samples in Ethiopia.

## ANNOUNCEMENT

Repeated cholera outbreaks have been consistently reported across Sub-Saharan Africa, including Ethiopia (1). In Ethiopia, an estimated 275,221 cases and 10,458 deaths have been reported annually. Between 2015 and 2024, the country experienced multiple transmission waves (2). The most significant wave emerged on the 10th of October, 2021, in the Bale Zone of the Oromia National Regional State. Between August 2022 and September 2023 alone, 23,449 cases and 317 deaths were reported, with a case fatality rate of 1.4% (3).

In this study, a total of 415 fecal samples were collected in Ethiopia in 2022. The samples were transported using Cary-Blair transport media at ambient temperature for microbiological analysis. Fecal samples were inoculated on blood agar (BAP, Oxoid), MacConkey agar (MAC, Oxoid), and thiosulfate citrate bile salt sucrose agar (TCBS, Oxoid) and incubated aerobically at 37°C for 24 hours. Of the 415 samples analyzed by culture, *V. cholerae* was confirmed in 33 samples through oxidase tests, string tests, and Gram staining. Genomic DNA was subsequently extracted from all 33 confirmed *V. cholerae* colonies using the QIAamp DNA Mini Kit (Qiagen, Hilden, Germany) and quantified using the Qubit 4.0 Fluorometer (Invitrogen, Cat. No. Q32854, Germany). Each DNA sample was then normalized to a final concentration of 4 nM prior to downstream applications. Library preparation of genomic DNA was processed using the Illumina DNA Prep kit (Cat. No. 20060060), which utilizes a bead-linked transposome to simultaneously fragment and tag DNA. Sequencing libraries were run on the Illumina NextSeq 550 platform (2 × 150 bp paired-end reads; Illumina, Singapore). Raw reads were evaluated using FASTQC v0.12.1**,** and low-quality bases and adapter sequences were trimmed with Trimmomatic v0.39. Reads were classified and screened for potential contaminants using kraken2 (V2) (4). High-quality short reads were aligned against the *V. cholerae* N16961 strain for both chromosome 1 (GenBank accession NC_002505.1) and chromosome 2 (GenBank accession NC_002506.1) utilizing the BWA-MEM algorithm. Mapping quality was evaluated using SAMtools stats (V1.21), achieving >30× depth and >98% genome coverage. Final genome assemblies revealed genomic metrics across 33 isolates. Finally, we quantified read count, genome coverage, average sequencing depth, and the GC contents to generate key genomic insights for circulating *V. cholerae* strains in Ethiopia. Notably, this represents the first draft genome sequence of *V. cholerae* from Ethiopia submitted to NCBI (Table 1).

**TABLE 1:** Draft genome sequence data of *Vibrio cholerae* from cholera outbreaks in Ethiopia^*a*^

Sample collection year	Sample ID	Sampling site	No of reads (2 × 150 reads)		Coverage (%)		Mean depth		GC content (%)		Accession number (raw reads)	Accession number (BioSample)
			Ch1	Ch2	Ch1	Ch2	Ch1	Ch2	Ch1	Ch2		
2022	ACH-3	AA	4,983,297	1,768,921	99.5	98.1	228.209	223.898	47.07	46.9	SRR32238366	SAMN46560025
2022	ACH-4	AA	1,173,497	414,861	99.5	98.1	53.2522	51.9992	47.07	46.9	SRR32238365	SAMN46560026
2022	ACH-5	AMH	2,651,089	926,703	99.5	98.0	120.689	116.603	47.07	46.9	SRR32238354	SAMN46560027
2022	ACH-6	AA	1,091,297	390,101	99.5	98.0	49.5062	48.8895	47.07	46.9	SRR32238343	SAMN46560028
2022	ACH-7	AMH	3,133,139	1,081,587	99.5	98.0	142.351	135.732	47.07	46.9	SRR32238338	SAMN46560029
2022	ACH-9	AA	3,679,383	1,301,267	99.5	98.1	167.369	163.495	47.07	46.9	SRR32238337	SAMN46560030
2022	ACH-10	AA	2,525,907	901,301	99.5	98.0	113.831	112.183	47.07	46.9	SRR32238336	SAMN46560031
2022	ACH-11	AMH	2,993,105	1,023,201	99.5	98.0	137.304	129.618	47.07	46.9	SRR32238335	SAMN46560032
2022	ACH-12	AMH	3,364,357	1,190,240	99.5	98.1	151.042	147.554	47.07	46.9	SRR32238334	SAMN46560033
2022	ACH-13	AA	2,450,469	879,363	99.5	98.1	107.28	106.313	47.07	46.9	SRR32238333	SAMN46560034
2022	ACH-16	AMH	6,471,870	2,180,580	99.5	98.1	296.804	276.256	47.07	46.9	SRR32238364	SAMN46560035
2022	ACH-17	AA	4,957,265	1,760,018	99.5	98.1	226.427	221.316	47.07	46.9	SRR32238363	SAMN46560036
2022	ACH-18	AA	4,701,797	1,642,834	99.5	98.1	214.244	206.774	47.07	46.9	SRR32238362	SAMN46560037
2022	ACH-19	AMH	7,274,467	2,471,464	99.5	98.1	330.65	310.436	47.07	46.9	SRR32238361	SAMN46560038
2022	ACH-20	AA	5,849,517	2,066,612	99.5	98.1	265.633	259.305	47.07	46.9	SRR32238360	SAMN46560039
2022	ACH-22	AMH	3,541,736	1,207,311	99.5	98.1	160.669	151.259	47.07	46.9	SRR32238359	SAMN46560040
2022	ACH-23	AMH	4,267,666	1,526,835	99.5	98.1	195.115	192.812	47.07	46.9	SRR32238358	SAMN46560041
2022	ACH-24	AMH	6,180,087	2,072,818	99.5	98.1	282.739	261.917	47.07	46.9	SRR32238357	SAMN46560042
2022	ACH-25	AA	5,488,871	1,947,836	99.5	98.1	249.804	244.836	47.07	46.9	SRR32238356	SAMN46560043
2022	ACH-26	AMH	15,803,696	5,468,562	99.5	98.1	720.111	688.336	47.07	46.9	SRR32238355	SAMN46560044
2022	ACH-28	AMH	5,643,846	1,946,908	99.5	98.1	256.369	244.24	47.07	46.9	SRR32238352	SAMN46560046
2022	ACH-31	AMH	5,219,525	1,780,032	99.5	98.1	236.364	222.741	47.07	46.9	SRR32238351	SAMN46560047
2022	ACH-32	AMH	6,160,279	2,097,270	99.5	98.1	277.205	260.754	47.07	46.9	SRR32238350	SAMN46560048
2022	ACH-33	AA	4,973,361	1,771,877	99.5	98.1	225.767	222.216	47.07	46.9	SRR32238349	SAMN46560049
2022	ACH-34	AMH	4,029,215	1,424,988	99.5	98.1	180.774	176.618	47.07	46.9	SRR32238348	SAMN46560050
2022	ACH-35	AA	1,632,144	584,853	99.5	98.1	70.6689	69.8969	47.07	46.9	SRR32238347	SAMN46560051
2022	ACH-37	AA	1,762,162	622,858	99.5	98.1	79.3458	77.5707	47.07	46.9	SRR32238346	SAMN46560052
2022	ACH-38	AMH	3,979,041	1,363,452	99.5	98.1	181.967	172.239	47.07	46.9	SRR32238345	SAMN46560053
2022	ACH-41	AMH	5,034,618	1,712,136	99.5	98.1	230.006	216.071	47.07	46.9	SRR32238344	SAMN46560054
2022	ACH-42	AMH	3,755,969	1,308,495	99.5	98.1	166.174	159.969	47.07	46.9	SRR32238342	SAMN46560055
2022	ACH-43	AMH	4,329,830	1,485,812	99.5	98.1	195.458	185.32	47.07	46.9	SRR32238341	SAMN46560056
2022	ACH-44	AMH	4,082,954	1,400,265	99.5	98.1	184.981	175.301	47.07	46.9	SRR32238340	SAMN46560057
2022	ACH-45	AMH	3,172,997	1,122,812	99.5	98.1	143.702	140.406	47.07	46.9	SRR32238339	SAMN46560058

^*a*^ NB: Ch1: chromosome 1, Ch2: chromosome 2, AA: Addis Ababa, AMH: Amhara.

## Data Availability

The *V. cholerae* O1 serogroup and sequence data were deposited in GenBank under BioProject SRA PRJNA1219533 with the accession numbers listed in Table 1.
